# [Expression of Concern] RUNX2 RNA interference inhibits the invasion of osteosarcoma

**DOI:** 10.3892/ol.2025.15243

**Published:** 2025-08-27

**Authors:** Heng Zeng, Xiaotao Xu

Oncol Lett 9: 2455–2458, 2015; DOI: 10.3892/ol.2015.3124

Following the publication of this paper, it was drawn to the Editor's attention by a concerned reader that, in Fig. 3, the cellular images for the “ConN” and “ConB” panels appeared to show an area of overlap, such that data which were intended to show the results from differently performed experiments had apparently been selected from the same original source. In addition, concerning the western blot data in Figs. 1 and 5, lanes 2–4 of the GAPDH panel in Fig. 1 looked strikingly similar to lanes 1–3 of the GAPDH panel of Fig. 5, even though these lanes were indicated to represent different experiments. Furthermore, an independent analysis of the data in this paper made by the Editorial Office showed that certain of the data in Fig. 3 were similar to data which appeared in a pair of subsequently published articles in other journals that were written by different authors at different research institutes.

The authors were contacted by the Editorial Office to offer an explanation for these apparent anomalies in the presentation of the data in this paper; however, up to this time, no response from them has been forthcoming. Owing to the fact that the Editorial Office has been made aware of potential issues surrounding the scientific integrity of this paper, we are issuing an Expression of Concern to notify readers of this potential problem while the Editorial Office continues to investigate this matter further.

